# Supplementary material for: Seroprevalence and lethality by SARS-CoV-2 in indigenous populations of Latin America and the Caribbean: a systematic review
Source: PeerJ. 2021 Dec 16;9:e12552. doi: 10.7717/peerj.12552 (PMC8684739; doi:10.7717/peerj.12552)
Supplement: Supplemental Information 1 [file peerj-09-12552-s001.docx]

**Appendix S1.** Search strategies

| **Database** | **PubMed** | |
| --- | --- | --- |
| **Strategy** | #1 | Coronavirus[Mesh] |
|  | #2 | Spike protein, SARS-CoV-2 [Supplementary Concept] |
|  | #3 | Severe Acute Respiratory Syndrome[Mesh] |
|  | #4 | COVID-19[Supplementary Concept] |
|  | #5 | Pediatric Multisystem Inflammatory Disease, COVID-19 Related[Supplementary Concept] |
|  | #6 | Corona Virus[tiab] |
|  | #7 | COVID-19[tiab] |
|  | #8 | COVID19*[tiab] |
|  | #9 | 2019-nCoV[tiab] |
|  | #10 | SARS-CoV-2[tiab] |
|  | #11 | SARS-CoV2[tiab] |
|  | #12 | SARSCoV2[tiab] |
|  | #13 | (Pneumonia[tiab] AND Wuhan[tiab] AND 2019[tiab]) |
|  | #14 | Coronavir*[tiab] |
|  | #15 | Coronovir*[tiab] |
|  | #16 | Virus Corona[tiab] |
|  | #17 | Corono Virus[tiab] |
|  | #18 | HCov*[tiab] |
|  | #19 | CV19*[tiab] |
|  | #20 | CV-19*[tiab] |
|  | #21 | N Cov*[tiab] |
|  | #22 | #1 OR #2 OR #3 OR #4 OR #5 OR #6 OR #7 OR #8 OR #9 OR #10 OR #11 OR #12 OR #13 OR #14 OR #15 OR #16 OR #17 OR #18 OR #19 OR #20 OR #21 |
|  | #23 | Indians, South American[Mesh] |
|  | #24 | Indians, Central American[Mesh] |
|  | #25 | American Indian*[tiab] |
|  | #26 | Amerindian*[tiab] |
|  | #27 | #23 OR #24 OR #25 OR #26 |
|  | #28 | Indigenous Peoples[Mesh] |
|  | #29 | Indigenous[tiab] |
|  | #30 | Indigene[tiab] |
|  | #31 | Ancestr*[tiab] |
|  | #32 | Indians[tiab] |
|  | #33 | First Nation[tiab] |
|  | #34 | Originary[tiab] |
|  | #35 | Aborigin*[tiab] |
|  | #36 | Native*[tiab] |
|  | #37 | #28 OR #29 OR #30 OR #31 OR #32 OR #33 OR #34 OR #35 OR #36 |
|  | #38 | (Latin America[Mesh] OR Latin America*[tiab] OR Latinamerica*[tiab] OR Latinoamerica*[tiab] OR Central America[Mesh] OR Central America*[tiab] OR Centroamerica*[tiab] OR Mesoamerica*[tiab] OR Meso America*[tiab] OR Middle America*[tiab] OR South America[Mesh] OR South America*[tiab] OR Southamerica*[tiab] OR Sudamerica*[tiab] OR "America del sur"[tiab] OR Caribbean Region[Mesh] OR Caribbean[tiab] OR Caribe*[tiab] OR West Indies[Mesh] OR West Indi*[tiab] OR Antill*[tiab] OR Patagoni*[tiab] OR Andes[tiab] OR Andean*[tiab] OR Amazon*[tiab] OR Argentin*[ad] OR Argentin*[tiab] OR Argentina[pl] OR Bolivia*[ad] OR Bolivia*[tiab] OR Bolivia[pl] OR Brazil*[ad] OR Brasil*[ad] OR Brazil*[tiab] OR Brasil*[tiab] OR Brazil[pl] OR Colombia*[ad] OR Colombia*[tiab] OR Colombia[pl] OR Chile*[ad] OR Chile*[tiab] OR Chile[pl] OR Ecuador*[ad] OR Ecuator*[ad] OR Ecuador*[tiab] OR Ecuador[pl] OR Guiana*[ad] OR Guiana*[tiab] OR French Guiana[pl] OR Guyan*[ad] OR Guyan*[tiab] OR Guyana[pl] OR Paraguay*[ad] OR Paraguay*[tiab] OR Paraguay[pl] OR Peru*[ad] OR Peru*[tiab] OR Peru[pl] OR Surinam*[ad] OR Surinam*[tiab] OR Suriname[pl] OR Uruguay*[ad] OR Uruguay*[tiab] OR Uruguay[pl] OR Venez*[ad] OR Venez*[tiab] OR Venezuela[pl] OR Belize*[ad] OR Belize*[tiab] OR Belize[pl] OR Costa Ric*[ad] OR Costarric*[ad] OR Costaric*[ad] OR Costa Ric*[tiab] OR Costarric*[tiab] OR Costaric*[tiab] OR Costa Rica[pl] OR Salvador*[ad] OR Salvador*[tiab] OR El Salvador[pl] OR Guatemal*[ad] OR Guatemal*[tiab] OR Guatemala[pl] OR Hondur*[ad] OR Hondur*[tiab] OR Honduras[pl] OR Nicaragu*[ad] OR Nicaragu*[tiab] OR Nicaragua[pl] OR Panam*[ad] OR Panam*[tiab] OR Panama[pl] OR Mexico[Mesh] OR Mexic*[ad] OR Mexic*[tiab] OR Mejic*[tiab] OR Mexico[pl] OR Cuba*[ad] OR Cuba*[tiab] OR Cuba[pl] OR Dominic*[ad] OR Dominic*[tiab] OR Dominican Republic[pl] OR Haiti*[ad] OR Haiti*[tiab] OR Haiti[pl] OR Jamaic*[ad] OR Jamaic*[tiab] OR Jamaica[pl] OR Puerto Rico[Mesh] OR Puerto Ric*[tiab] OR Puertorric*[tiab] OR Puertoric*[tiab]) |
|  | #39 | #38 AND #39 |
|  | #40 | #27 OR #40 |
|  | #41 | #22 AND #41 |

| **Database** | **Embase** | |
| --- | --- | --- |
| **Strategy** | #1 | 'coronavirinae'/exp |
|  | #2 | 'sars coronavirus'/exp |
|  | #3 | 'severe acute respiratory syndrome'/exp |
|  | #4 | 'coronavirus disease 2019'/exp |
|  | #5 | (corona NEAR/1 virus):ti,ab |
|  | #6 | (corono NEAR/1 virus):ti,ab |
|  | #7 | 'covid 19':ti,ab |
|  | #8 | covid19*:ti,ab |
|  | #9 | '2019 ncov':ti,ab |
|  | #10 | 'sars cov 2':ti,ab |
|  | #11 | 'sars cov2':ti,ab |
|  | #12 | sarscov2:ti,ab |
|  | #13 | pneumonia:ti,ab AND wuhan:ti,ab AND 2019:ti,ab |
|  | #14 | coronavir*:ti,ab |
|  | #15 | coronovir*:ti,ab |
|  | #16 | hcov*:ti,ab |
|  | #17 | cv19*:ti,ab |
|  | #18 | 'cv 19*':ti,ab |
|  | #19 | 'n cov*':ti,ab |
|  | #20 | #1 OR #2 OR #3 OR #4 OR #5 OR #6 OR #7 OR #8 OR #9 OR #10 OR #11 OR #12 OR #13 OR #14 OR #15 OR #16 OR #17 OR #18 OR #19 |
|  | #21 | 'american indian'/exp |
|  | #22 | (american NEAR/1 indian*):ti,ab |
|  | #23 | amerindian*:ti,ab |
|  | #24 | #21 OR #22 OR #23 |
|  | #25 | 'indigenous people'/exp |
|  | #26 | indigenous:ti,ab |
|  | #27 | indigene:ti,ab |
|  | #28 | ancestr*:ti,ab |
|  | #29 | indians:ti,ab |
|  | #30 | (first NEAR/1 nation):ti,ab |
|  | #31 | originary:ti,ab |
|  | #32 | aborigin*:ti,ab |
|  | #33 | native*:ti,ab |
|  | #34 | #25 OR #26 OR #27 OR #28 OR #29 OR #30 OR #31 OR #32 OR #33 |
|  | #35 | 'south and central america'/exp OR ((latin NEAR/1 america*):ti,ab) OR latinamerica*:ti,ab OR latinoamerica*:ti,ab OR hispanoamerica:ti,ab OR iberoamerica*:ti,ab OR ((ibero NEAR/1 americ*):ti,ab) OR panamerica*:ti,ab OR ((south NEAR/1 america*):ti,ab) OR southamerica*:ti,ab OR sudamerica*:ti,ab OR (america:ti,ab AND del:ti,ab AND sur:ti,ab) OR ((central NEAR/1 america*):ti,ab) OR centroamerica*:ti,ab OR mesoamerica*:ti,ab OR ((meso NEAR/1 america*):ti,ab) OR ((middle NEAR/1 america*):ti,ab) OR 'caribbean'/exp OR 'caribbean islands'/exp OR caribbean*:ti,ab OR caribe*:ti,ab OR ((west NEAR/1 indi*):ti,ab) OR antill*:ti,ab OR 'american indian'/exp OR amerindian*:ti,ab OR indians:ti,ab OR ((native NEAR/1 america*):ti,ab) OR patagoni*:ti,ab OR andes:ti,ab OR andean*:ti,ab OR amazon*:ti,ab OR 'argentina'/exp OR argentin*:ti,ab OR 'bolivia'/exp OR bolivia*:ti,ab OR 'brazil'/exp OR brazil*:ti,ab OR brasil*:ti,ab OR 'colombia'/exp OR colombia*:ti,ab OR 'chile'/exp OR chile*:ti,ab OR 'ecuador'/exp OR ecuador*:ti,ab OR 'french guiana'/exp OR guiana*:ti,ab OR 'guyana'/exp OR guyan*:ti,ab OR 'paraguay'/exp OR paraguay*:ti,ab OR 'peru'/exp OR peru*:ti,ab OR 'suriname'/exp OR surinam*:ti,ab OR 'uruguay'/exp OR uruguay*:ti,ab OR 'venezuela'/exp OR venez*:ti,ab OR 'belize'/exp OR beliz*:ti,ab OR 'costa rica'/exp OR 'costa rica':ti,ab OR costarric*:ti,ab OR costaric*:ti,ab OR 'el salvador'/exp OR salvador*:ti,ab OR 'guatemala'/exp OR guatemal*:ti,ab OR 'honduras'/exp OR hondur*:ti,ab OR 'nicaragua'/exp OR nicaragu*:ti,ab OR 'panama'/exp OR panam*:ti,ab OR 'mexico'/exp OR mexic*:ti,ab OR mejic*:ti,ab OR 'cuba'/exp OR cuba*:ti,ab OR 'dominican republic'/exp OR dominica*:ti,ab OR 'haiti'/exp OR haiti*:ti,ab OR 'jamaica'/exp OR jamaic*:ti,ab OR 'puerto rico'/exp OR ((puerto NEAR/1 ric*):ti,ab) OR puertoric*:ti,ab OR puertorric*:ti,ab |
|  | #36 | #34 AND #35 |
|  | #37 | #24 OR #36 |
|  | #38 | #20 AND #37 |

| **Database** | **CINAHL** | |
| --- | --- | --- |
| **Strategy** | #1 | (MH "Coronavirus+") |
|  | #2 | (MH "Coronavirus Infections+") |
|  | #3 | (MH "COVID-19") |
|  | #4 | TI (Coron* N1 Virus) OR AB (Coron* N1 Virus) |
|  | #5 | TI COVID-19* OR AB COVID-19* |
|  | #6 | TI COVID19* OR AB COVID19* |
|  | #7 | TI 2019-nCoV OR AB 2019-nCoV |
|  | #8 | TI SARS-CoV-2 OR AB SARS-CoV-2 |
|  | #9 | TI SARS-CoV2 OR AB SARS-CoV2 |
|  | #10 | TI SARSCoV2 OR AB SARSCoV2 |
|  | #11 | TI Coronavir* OR AB Coronavir* |
|  | #12 | TI Coronovir* OR AB Coronovir* |
|  | #13 | TI HCov* OR AB HCov* |
|  | #14 | TI CV19* OR AB CV19* |
|  | #15 | TI CV19* OR AB CV19* |
|  | #16 | TI CV-19* OR AB CV-19* |
|  | #17 | TI N-Cov* OR AB N-Cov* |
|  | #18 | #1 OR #2 OR #3 OR #4 OR #5 OR #6 OR #7 OR #8 OR #9 OR #10 OR #11 OR #12 OR #13 OR #14 OR #15 OR #16 OR #17 |
|  | #19 | TI (American N1 Indian*) OR AB (American N1 Indian*) |
|  | #20 | TI Amerindian* OR AB Amerindian* |
|  | #21 | (MM "Native Americans") |
|  | #22 | (MH "Indigenous Peoples+") |
|  | #23 | TI Indigenous OR AB Indigenous |
|  | #24 | TI Native* OR AB Native* |
|  | #25 | TI Ancestr* OR AB Ancestr* |
|  | #26 | TI Indians* OR AB Indians |
|  | #27 | #19 OR #20 OR #21 OR #22 OR #23 OR #24 OR #25 OR #26 |
|  | #28 | TI ((Latin N1 America*) OR Latinamerica* OR Latinoamerica* OR Latin* OR Hispanic Americans OR Iberoamerica* OR Ibero Americ* OR Panamerican* OR Central America* OR Centroamerica* OR Mesoamerica* OR Meso America* OR Middle America* OR South America* OR Southamerica* OR Sudamerica* OR America del sur OR Caribbean OR Caribe* OR West Indi* OR Antill* OR Amerindian* OR Indians OR American Indian* OR Native America* OR Patagoni* OR Andes OR Andean* OR Amazon* OR Argentin* OR Bolivia* OR Brazil* OR Brasil* Colombia* OR Colombia* OR Colombia OR Chile* OR Ecuador* OR Guiana* OR Guyan* OR Guyan* OR Paraguay* OR Paraguay* OR Peru* OR Surinam* OR Surinam* OR Uruguay* OR Venez* OR Belize* OR Costa Ric* OR Costarric* OR Costaric* OR Costa Ric* OR Costarric* OR Salvador* OR Salvador* OR Salvador OR Guatemal* OR Guatemal* OR Guatemala OR Hondur* OR Nicaragu* Panam* OR Mexic* OR Cuba* OR Dominic* OR Dominic* OR Haiti* OR Jamaic* OR Puerto Ric* OR Puertorric* OR Puertoric*) |
|  | #29 | AB ((Latin N1 America*) OR Latinamerica* OR Latinoamerica* OR Latin* OR Hispanic Americans OR Iberoamerica* OR Ibero Americ* OR Panamerican* OR Central America* OR Centroamerica* OR Mesoamerica* OR Meso America* OR Middle America* OR South America* OR Southamerica* OR Sudamerica* OR America del sur OR Caribbean OR Caribe* OR West Indi* OR Antill* OR Amerindian* OR Indians OR American Indian* OR Native America* OR Patagoni* OR Andes OR Andean* OR Amazon* OR Argentin* OR Bolivia* OR Brazil* OR Brasil* Colombia* OR Colombia* OR Colombia OR Chile* OR Ecuador* OR Guiana* OR Guyan* OR Guyan* OR Paraguay* OR Paraguay* OR Peru* OR Surinam* OR Surinam* OR Uruguay* OR Venez* OR Belize* OR Costa Ric* OR Costarric* OR Costaric* OR Costa Ric* OR Costarric* OR Salvador* OR Salvador* OR Salvador OR Guatemal* OR Guatemal* OR Guatemala OR Hondur* OR Nicaragu* Panam* OR Mexic* OR Cuba* OR Dominic* OR Dominic* OR Haiti* OR Jamaic* OR Puerto Ric* OR Puertorric* OR Puertoric*) |
|  | #30 | #28 OR #29 |
|  | #31 | #18 AND #27 AND #30 |

| **Database** | **Web of Science**  Indexes=SCI-EXPANDED, SSCI, A&HCI, ESCI. Period of time = All years | |
| --- | --- | --- |
| **Strategy** | #1 | TS=Coronavirus |
|  | #2 | TS=Severe Acute Respiratory Syndrome |
|  | #3 | TS=Pediatric Multisystem Inflammatory Disease |
|  | #4 | TS=COVID-19 |
|  | #5 | TI=COVID-19* |
|  | #6 | TI=COVID19* |
|  | #7 | TI=2019-nCoV |
|  | #8 | TI=SARS-CoV-2 |
|  | #9 | TI=SARSCoV2 |
|  | #10 | TI=Coronavir* |
|  | #11 | TI=Coronovir* |
|  | #12 | TI=(Virus NEAR/1 Coron*) |
|  | #13 | TI=HCov* |
|  | #14 | TI=CV19* |
|  | #15 | TI=CV-19* |
|  | #16 | TI=N-Cov* |
|  | #17 | #1 OR #2 OR #3 OR #4 OR #5 OR #6 OR #7 OR #8 OR #9 OR #10 OR #11 OR #12 OR #13 OR #14 OR #15 OR #16 |
|  | #18 | TI=(American NEAR/1 Indian*) |
|  | #19 | TS=Amerindians |
|  | #20 | TI=Amerindian* |
|  | #21 | TS=Indigenous People |
|  | #22 | TI=Indigenous |
|  | #23 | TI=Native* |
|  | #24 | TI=Ancestr* |
|  | #25 | TI=Indians |
|  | #26 | #18 OR #19 OR #20 OR #21 OR #22 OR #23 OR #24 OR #25 |
|  | #27 | TS=Latin America |
|  | #28 | TI=(Latin NEAR/1 America*) |
|  | #29 | TI=Latinamerica* |
|  | #30 | TI=Latinoamerica* |
|  | #31 | TS=Central America |
|  | #32 | TI=(Central NEAR/1 America*) |
|  | #33 | TI=Centroamerica* |
|  | #34 | TI=Mesoamerica* |
|  | #35 | TI=Meso America* |
|  | #36 | TI=Middle America* |
|  | #37 | TS=South America |
|  | #38 | TI=(South NEAR/1 America*) |
|  | #39 | TI=Southamerica* |
|  | #40 | TI=Sudamerica* |
|  | #41 | TS=Caribbean Region |
|  | #42 | TI=Caribbean |
|  | #43 | TS=West Indies |
|  | #44 | TI=(West Indi*) |
|  | #45 | TI=Antill* |
|  | #46 | TI=Patagoni* |
|  | #47 | TI=Andes |
|  | #48 | TI=Andean* |
|  | #49 | TI=Amazon* |
|  | #50 | TI=(Argentin* OR Bolivia* OR Brazil* OR Brasil* Colombia* OR Colombia* OR Colombia OR Chile* OR Ecuador* OR Guiana* OR Guyan* OR Guyan* OR Paraguay* OR Paraguay* OR Peru* OR Surinam* OR Surinam* OR Uruguay* OR Venez* OR Belize* OR (Costa NEAR/1 ric*) OR Costarric* OR Costaric* OR Costa Ric* OR Costarric* OR Salvador* OR Salvador* OR Guatemal* OR Guatemal* OR Guatemala OR Hondur* OR Nicaragu* OR Panam* OR Mexic* OR Cuba* OR Dominic* OR Dominic* OR Haiti* OR Jamaic* OR (Puerto NEAR/1 Ric*) OR Puertorric* OR Puertoric*) |
|  | #51 | TI=Mexic* |
|  | #52 | #51 OR #50 OR #49 OR #48 OR #47 OR #46 OR #45 OR #44 OR #43 OR #42 OR #41 OR #40 OR #39 OR #38 OR #37 OR #36 OR #35 OR #34 OR #33 OR #32 OR #31 OR #30 OR #29 OR #28 OR #27 |
|  | #53 | #52 AND #26 AND #17 |

| **Database** | **Scopus** | |
| --- | --- | --- |
| **Strategy** | #1 | TITLE-ABS-KEY (Coronavirus) |
|  | #2 | TITLE-ABS-KEY ("SARS-CoV-2 Spike protein") |
|  | #3 | TITLE-ABS-KEY ("Severe Acute Respiratory Syndrome") |
|  | #4 | TITLE-ABS-KEY ("COVID-19") |
|  | #5 | TITLE-ABS-KEY ("COVID-19 Related Pediatric Multisystem Inflammatory Disease") |
|  | #6 | TITLE-ABS-KEY ("Corona Virus") |
|  | #7 | TITLE-ABS-KEY ("COVID 19") |
|  | #8 | TITLE-ABS-KEY (COVID19*) |
|  | #9 | TITLE-ABS-KEY ("2019-nCoV") |
|  | #10 | TITLE-ABS-KEY ("SARS-CoV-2") |
|  | #11 | TITLE-ABS-KEY ("SARS-CoV2") |
|  | #12 | TITLE-ABS-KEY (SARSCoV2) |
|  | #13 | TITLE-ABS-KEY (Pneumonia AND Wuhan AND 2019) |
|  | #14 | TITLE-ABS-KEY (Coronavir*) |
|  | #15 | TITLE-ABS-KEY (Coronovir*) |
|  | #16 | TITLE-ABS-KEY ("Virus Corona") |
|  | #17 | TITLE-ABS-KEY ("Corono Virus") |
|  | #18 | TITLE-ABS-KEY (HCov*) |
|  | #19 | TITLE-ABS-KEY (CV19*) |
|  | #20 | TITLE-ABS-KEY (CV-19*) |
|  | #21 | TITLE-ABS-KEY (N-Cov*) |
|  | #22 | #1 OR #2 OR #3 OR #4 OR #5 OR #6 OR #7 OR #8 OR #9 OR #10 OR #11 OR #12 OR #13 OR #14 OR #15 OR #16 OR #17 OR #18 OR #19 OR #20 OR #21 |
|  | #23 | TITLE-ABS-KEY ("South American Indians") |
|  | #24 | TITLE-ABS-KEY ("Central American Indians") |
|  | #25 | TITLE-ABS-KEY (American Indian*) |
|  | #26 | TITLE-ABS-KEY (Amerindian*) |
|  | #27 | #23 OR #24 OR #25 OR #26 |
|  | #28 | TITLE-ABS-KEY ("Indigenous Peoples") |
|  | #29 | TITLE-ABS-KEY (Indigenous) |
|  | #30 | TITLE-ABS-KEY (Indigene) |
|  | #31 | TITLE-ABS-KEY (Ancestr*) |
|  | #32 | TITLE-ABS-KEY (Indians) |
|  | #33 | TITLE-ABS-KEY ("First Nation") |
|  | #34 | TITLE-ABS-KEY (Originary) |
|  | #35 | TITLE-ABS-KEY (Aborigin*) |
|  | #36 | TITLE-ABS-KEY (Native*) |
|  | #37 | #28 OR #29 OR #30 OR #31 OR #32 OR #33 OR #34 OR #35 OR #36 |
|  | #38 | TITLE-ABS-KEY ("latin america" OR "Latinoamerica" OR latin* OR "central america" OR "Centroamerica" OR "south America" OR sudamerica OR caribbean OR caribe* OR "west indies" OR antill* OR patagoni* OR andes OR andean OR amazon OR "Puerto rico" OR puertoric* OR puertorric* OR jamaica OR jamaic* OR haiti OR haiti* OR "dominican republic" OR dominica* OR cuba OR cuba* OR mexico OR mexic* OR mejic* OR panama OR panam* OR nicaragua OR nicaragu* OR honduras OR hondur* OR guatemala OR guatemal* OR "el Salvador" OR salvador* OR "costa rica" OR costarric* OR costaric* OR belize OR beliz* OR venezuela OR venez* OR uruguay OR uruguay* OR suriname OR surinam* OR peru OR peru* OR paraguay OR paraguay* OR guyana OR guyan* OR "french guiana" OR guiana* OR guayan* OR ecuador OR ecuador* OR chile OR chile* OR colombia OR colombia* OR brazil OR brazil* OR brasil* OR bolivia OR bolivia* OR argentina OR argentin*) |
|  | #39 | #37 AND #38 |
|  | #40 | #27 OR #39 |
|  | #41 | #22 AND #40 |

| **Database** | **LILACS** | |
| --- | --- | --- |
| **Strategy** | #1 | (MH Coronavirus OR MH Coronavirus Infections OR Corona OR COVID-19$ OR COVID19$ OR 2019-nCoV OR SARS-CoV-2 OR SARS-CoV2 OR SARSCoV2 OR Coronavir$ OR Coronovir$ OR HCov$ OR CV19$ OR CV-19$ OR N-Cov$) AND (MH Indians, South American OR MH Indians, Central American OR Indian$ OR Amerindian$ OR Indios OR MH Indigenous Peoples OR Indigen$ OR Native$ OR Nativo$ OR Ancestr$ OR Originar$ OR Aborig$ OR Native$ OR Nativo$) [Words] |
