# Supplementary material for: Seroprevalence and lethality by SARS-CoV-2 in indigenous populations of Latin America and the Caribbean: a systematic review
Source: PeerJ. 2021 Dec 16;9:e12552. doi: 10.7717/peerj.12552 (PMC8684739; doi:10.7717/peerj.12552)
Supplement: Supplemental Information 3 [file peerj-09-12552-s003.docx]

Systematic Review and/or Meta Analysis Rationale.

Dear PeerJ readers,

This Systematic Review was conducted in order to better our understanding of the distribution and lethality of COVID-19 among the indigenous population of Latin America and the Caribbean (LAC). Even though LAC concentrated some of the highest numbers of cases and deaths due to COVID-19, a Systematic Review has yet to be conducted to measure the seroprevalence and lethality of the COVID-19 pandemic on this historically vulnerable population which represents 8% of the total population of LAC.

In spite of the fact that the variability in the reporting of results and limited number of studies conducted among indigenous people of LAC did not allow a meta-analysis to be carried out. We hope this study will contribute to improve our understanding of the distribution of COVID-19 among the indigenous population of LAC to and help generate strategies oriented towards better allocation of health resources in this population.

Regards,

Antonio Barrenechea-Pulache

On behalf of all the co-authors
